# Posterior vertebral column resection for correction of thoracolumbar kyphosis after failed anterior instrumented fusion

**DOI:** 10.1097/MD.0000000000020982

**Published:** 2020-07-17

**Authors:** Jian Lu, Zhe-Hao Dai, Hai-Sheng Li, Yi-Jun Kang, Fei Chen

**Affiliations:** a Department of Spine Surgery, The Second Xiangya Hospital of Central South University, Changsha; b Department of Orthopaedics, The Affiliated Suzhou Science & Technology Town Hospital of Nanjing Medical University, Suzhou, China; c Department of Orthopaedics E, Aarhus University Hospital, Aarhus, Denmark.

**Keywords:** anterior fusion, graft failure, posterior corrective osteotomy, thoracolumbar kyphosis

## Abstract

To explore the effectiveness of posterior vertebral column resection for failed thoracolumbar anterior instrumented fusion.

Ten patients with anterior fusion with refractory pain, progressive neurological deficits, and kyphosis were recruited. Anterior removal of the implant and posterior vertebral column resection were performed. The mean operating time, intraoperative blood loss, kyphosis angle, visual analog scale pain score, Oswestry disability index, bone fusion time, and complications were assessed in a minimum 18-month follow-up.

The mean operating time was 323.5 ± 63.6 minutes, with a mean blood loss of 1189 ± 253.4 mL. The mean preoperative kyphosis angle of 54.6° ± 8.0° immediately decreased to 4.8° ± 1.5° after revision surgery and eventually to 6.8° ± 1.3° at the final follow-up. The mean bone fusion time was 6.8 ± 1.2 months. All patients had satisfactory sagittal and coronal balance with no implant failure at the last follow-up. The average visual analog scale score was 6.2 ± 1.0 preoperatively, and it decreased to 2.6 ± 0.5 at the last follow-up. No patient suffered from neurological deterioration. The Oswestry disability index decreased from 39.8% ± 4.6% preoperatively to 24.5% ± 4.7% at the final follow-up. Complications occurred in 4 patients: 3 experienced tearing of the lung, and 1 had a superficial wound infection.

Anterior removal of the implant and posterior vertebral column resection constituted a safe and effective revision surgery for patients with prior anterior fusion with rigid postsurgical deformities.

## 1 Introduction

Anterior thoracolumbar spine surgery is typically indicated for the restoration of structural stability and/or decompression of a neural element secondary to trauma, tumor, or infection. The benefits of the anterior approach include direct access to the ventral pathology, restoration of the spine's load-bearing column, and more complete and direct decompression of neural elements.^[1–5]^ After decompression or debridement, reconstruction of the defect with a bone graft is often required to ensure stability. During the process of bone graft incorporation, the graft is resorbed, replaced, and remodeled, and a smaller and denser mass is formed.^[6,7]^ The mutation that occurs during the consolidation process is an important indicator of successful fusion.^[2,8]^ The resulting loss of volume and length is accompanied by a loss of correction. In a study by Rajasekaran and Soundarapandian,^[5]^ only 41% of spinal tuberculosis patients treated with anterior fusion alone achieved stable structural maintenance of the bone graft, with the other 59% experiencing bone graft failure due to slippage, fracture, absorption, or subsidence.

To avoid such complications and allow early mobilization, additional anterior or posterior instrumentation may be attempted.^[8–11]^ However, a certain degree of correctional loss persists and cannot be prevented by additional instrumentation. Briem et al^[12]^ incorporated a plate for additional anterior stabilization and observed a correctional loss of 4.2° over the 12-month study period. In their study, 6.6% of cases showed strut graft failure, and full autograft integration was achieved in only 77% of the cases. Kim et al^[13]^ reported that a correction of 67.7% (11.3°) was achieved initially by anterior instrumentation in 21 patients with Pott disease, but 83% of the correction (9.4°) had been lost at the last follow-up. In another study,^[14]^ anterior decompression and fusion supplemented by the Kaneda device was performed for 20 patients with thoracolumbar burst fractures. The average correction of kyphosis was approximately 50% postoperatively, with a loss of approximately 50% of correction at the follow-up.^[14]^

Most correction losses are mild and acceptable, but patients with a kyphotic deformity of 30° or greater are at increased risk for chronic pain in the kyphotic region.^[15]^ As the gravity line shifts forward, the paraspinal musculature is weakened, resulting in progressive degenerative changes at adjacent levels and, occasionally, the development of progressive neurologic deficits.^[3,15–17]^ Compensatory hyperextension of the lower lumbar spine has been linked to a higher incidence of low-back pain, degenerative facet arthritis, and painful spondylolysis.^[18,19]^ Moreover, severe kyphosis can be associated with cosmetic and psychological problems in these patients.

The treatment of postsurgical kyphosis poses distinct challenges to spinal surgeons. In comparison to that of kyphosis affiliated with other etiologies, correction of postsurgical kyphosis via an anterior approach is complicated by the need to remove the anterior implant as well as by any distorted anatomy, visceral and vessel adhesions anterior to the spinal column, ventral scarring or tethering to the posterior longitudinal ligament or dura, and involvement of a collapsed and/or subsided strut graft in the apical fusion mass. In the present study, we reviewed 10 cases of kyphotic deformity with previous failed anterior surgery and attempted to answer the following questions: “When should revision surgery be performed?” “How should the revision surgery be done?” and “Why did prior surgery fail and what can be done to avoid future failures?” Our indications for revision surgery included pain refractory to conservative treatment, a progressive neurological deficit, and kyphosis.

## 2 Materials and methods

### 2.1 Patient population

Ten patients with kyphosis after anterior thoracolumbar instrumented fusion were admitted to our department from October 2009 to December 2014. Following posterior implant failure and kyphosis progression, patients were treated with removal of the failed posterior implant, anterior decompression, and instrumented stabilization as revision surgery. The patients primarily complained of intractable pain, deteriorating neurological deficits, and progressive deformities. Back pain severity was assessed using the visual analog scale, and clinical outcome was represented by the Oswestry disability index.

### 2.2 Diagnostic evaluation

Computed tomography with 3-dimensional reconstruction imaging was carried out routinely to define the distorted bony anatomy and adjacent structures and to determine the range of kyphosis and fusion status postoperatively. Magnetic resonance imaging was applied to evaluate the site, direction, and nature of compression as well as the spinal cord status.

### 2.3 Operative procedure

Under general anesthesia, the patient was first placed on an appropriate surgical frame in the lateral decubitus position with the previously operated side facing upwards. The existing scar was resected, and the soft tissue was reopened layer-by-layer. Because fibrosis was typically present in the previously operated area, meticulous dissection was required to avoid inadvertent injury. Following identification of the surrounding structures, the anterior implant was exposed along its full length and removed. A suction drain was placed at the resection site, and the wound was closed. Next, the patient was placed in the prone position. A posterior midline skin incision exposed at least 2 levels above and below the deformity, and pedicle screws were placed at corresponding levels on both sides. Laminectomies were performed to allow ample space for the dural sac and neural elements. Wide lateral dissection to the transverse processes was performed to facilitate vertebral body resection. In the thoracic spine, the corresponding rib heads were removed, and extrapleural dissection was performed via a costotransversectomy approach to expose the lateral margins of the apical fusion mass. To maintain spinal stability, a temporary rod (contoured to accommodate the deformity) was applied. The pedicle of the affected level was excised, and the nerve roots were gently retracted to make room for the osteotomy. Careful subperiosteal dissection was performed on 1 side (opposite the initial anterior stabilization) following the lateral wall of the vertebral body until the anterior aspect was easily palpable. Under direct visualization, the pedicles and lateral portions of the apical fusion mass were removed using a small osteotome, a high-speed burr, and/or rongeurs. A thin shell of bone at the anterior part, together with the anterior longitudinal ligament to protect translation, was left intact during removal of the body. The anterior apical fusion mass, including the collapsed or/and subsided bone graft, as well as any residual vertebral body, disc tissue, and surrounding callus, were removed. During the osteotomy procedure, the surgeon took care to always work within bony confines without straying into soft tissue. The last step of the osteotomy, removal of the lateral bony wall at the original instrumentation side, decreased the risk of inadvertent injuries. After completing the resection on 1 side, another temporary rod was inserted and securely locked to the screws. The first temporary rod was then removed to allow resection of the remaining anterior fusion mass on the other side. Deformity correction was performed by replacing the temporary rods with rods precontoured to the corrected shape. The deformity was gradually corrected with repeated additional rod compression. During the correction procedure, the surgeon continuously viewed the circumferentially decompressed spinal cord. If any pressure on the spinal cord was present at the margins of the laminectomy or reduction in dural pulsations, extension of the laminectomy was advisable. An appropriately sized tricortical iliac crest autograft was placed in the anterior defect to prevent excessive shortening of the spinal column, and a final compression was performed to secure its stabilization. To further stabilize the spine, arthrodesis of the spine, equal to the length of the instrumentation, was performed. Bone grafts obtained from decompression were placed in the remaining gap in the anterior column. Closed suction drains were inserted at the resection site, and the surgical wound was closed layer-by-layer. All surgeries were performed with somatosensory-evoked potential monitoring.

### 2.4 Postoperative care

The wound drains were removed once the output had decreased to less than 50 mL/24 h. Seventy-two hours after surgery, the patients were allowed to sit up in bed. Mobilization was allowed after the first postoperative week. The patients wore a thoracolumbar orthosis for the next 2 months and were followed at regular intervals for clinical and radiological evaluations.

### 2.5 Statistical analysis

All analyses were conducted using SPSS statistical software (version 16.0, IBM

Corp, Armonk, NY). Values were calculated as the mean ± standard deviation. Student *t* test was used to estimate the change in data by the comparison of postoperative, last follow-up, and preoperative data. All statistical tests were 2-sided, and statistical significance was set at *P* < .05.

### 2.6 Ethical review and patient consent

This study was approved by the ethics committee of the 2nd Xiangya Hospital of Central South University. Written informed consent was obtained from the patients for publication of this study and any accompanying images.

## 3 Results

The 10 patients reviewed in this study included 6 males and 4 females, with a mean age of 38.7 ± 9.2 years old (range, 24–56 years) and an average kyphosis angle of 54.6° ± 8.0° (range, 45°–68°). The average interval between anterior surgery and revision surgery was 58.9 ± 27.2 months (range, 32–115 months). Five patients had posttraumatic thoracolumbar kyphosis, and 5 had thoracolumbar tuberculosis in the initial surgery. Among the 5 patients with posttraumatic kyphosis, 2 had thoracolumbar fracture primarily treated with posterior instrumentation and laminectomy for decompression. All anterior implants were left sided. Based on the classification system of Frankel, the preoperative neurological status was grade E in 6 patients, grade D in 2 patients, and grade C in 2 patients. The demographic data for each patient are provided in Table 1.

**Table 1:**
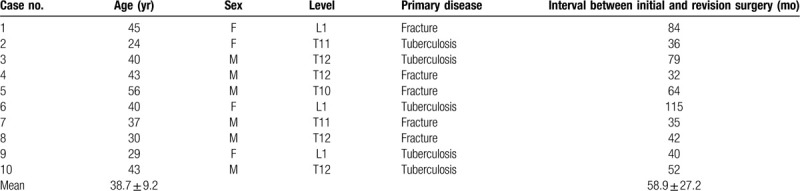
Patient demographics.

### 3.1 Surgical results and complications

The mean operating time for revision surgery was 323.5 ± 63.6 minutes (range, 245–420 minutes), and the average intraoperative blood loss was 1189 ± 253.4 mL (range, 850–1550 mL). Operative complications occurred in 4 patients (40%). Tearing of the lung occurred in 3 patients, and a superficial wound infection developed in 1 patient. Each lung tear at the site of pleural adhesion was initially repaired with a running 4-0 Prolene suture and then checked by reinflation of the lung to a pressure of 30 to 35 cmH_2_O after irrigation with saline warmed to 37°C into the thoracic cavity. No bubbling was observed, which confirmed that there was no air leakage. Closed chest drainage was established and removed when no air leaked from the thoracic cavity. The wound infection was treated with parenteral antibiotic therapy and managed successfully. No subsequent major complications occurred.

### 3.2 Clinical and radiographic results

**Figure 1:**
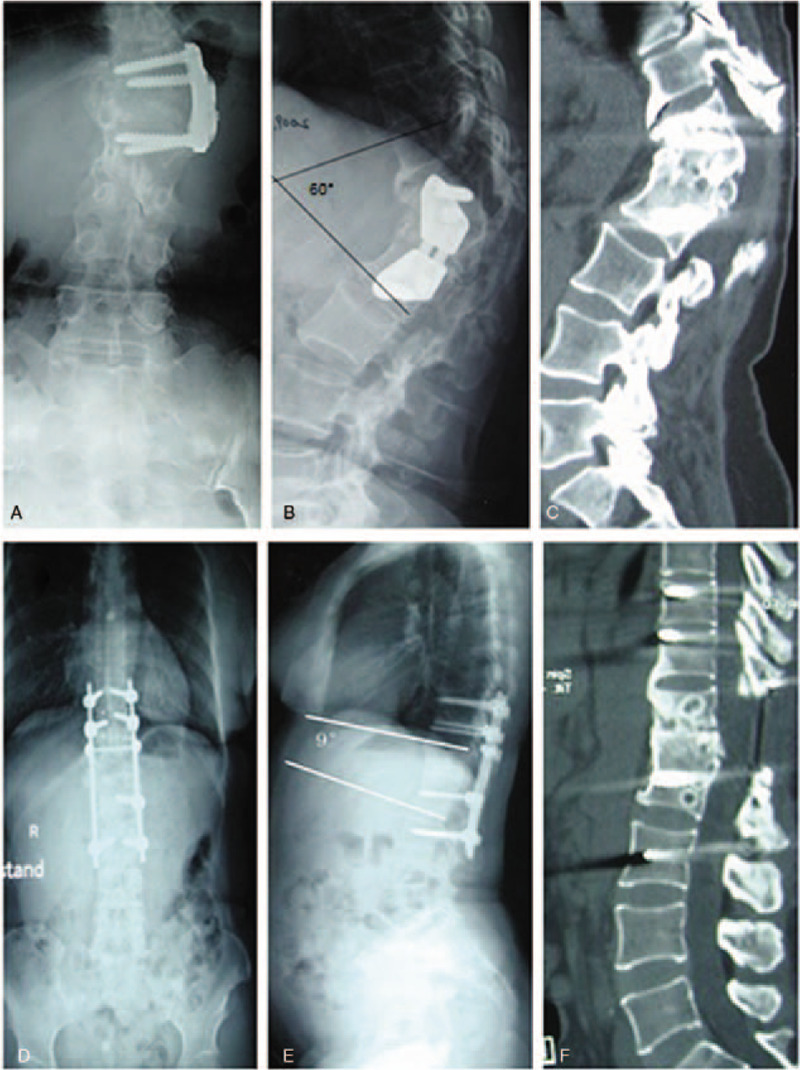
A 45-yr-old woman experienced failure after instrumentation with a Harrington rod and laminectomy for decompression because of an L1 fracture in 1996 and was treated by removal of the failed posterior instrumentation, anterior L1 corpectomy, strut grafting, and instrumentation with a ventro fix from T12-L2 in 2001 (radiographs were lost). In 2009, the patient's main complaints were increasing and intolerable back pain and numbness of the lower limbs. (A and B) X-ray images showed a thoracolumbar kyphosis of 60° after anterior instrumented fusion. (C) CT scan showing bony fusion between T12 and L1. (D and E) Radiographs showing kyphosis was corrected to 9° and maintained during the 2-yr follow-up. (F) CT scan showing bony fusion in the resection site and the trajectory of screws of the anterior implant. CT = computed tomography.

**Figure 2:**
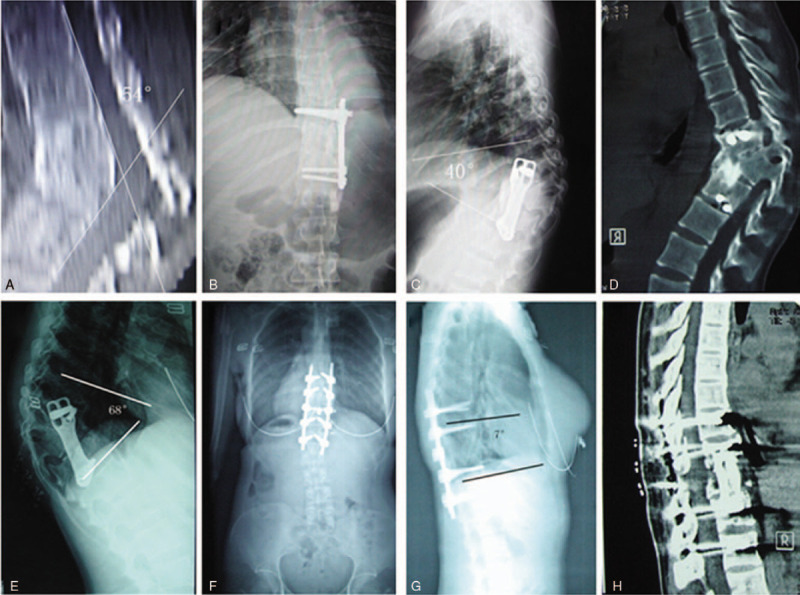
A 24-yr-old woman was treated with T11 corpectomy, strut grafting, and anterior instrumentation from T10-L1 due to thoracolumbar tuberculosis. Her main complaint was increasing kyphosis at 2 yr after the first operation. (A) Reconstructed CT image showing thoracolumbar kyphosis of 54°. (B and C) Postoperative X-ray showing correction of thoracolumbar kyphosis to 40°. (D) CT scan from 2-yr follow-up showing bony fusion of the strut and that the lower screw was retro pulsed. (E) X-ray image from 2-yr follow-up showing progression of thoracolumbar kyphosis to 68°. (F, G, and H) Postoperative X-ray images after the revision surgery showing fixation of the posterior implant in T8-L1, correction of thoracolumbar kyphosis to 7°, and maintenance of this angle the 2-yr follow-up. CT = computed tomography.

**Figure 3:**
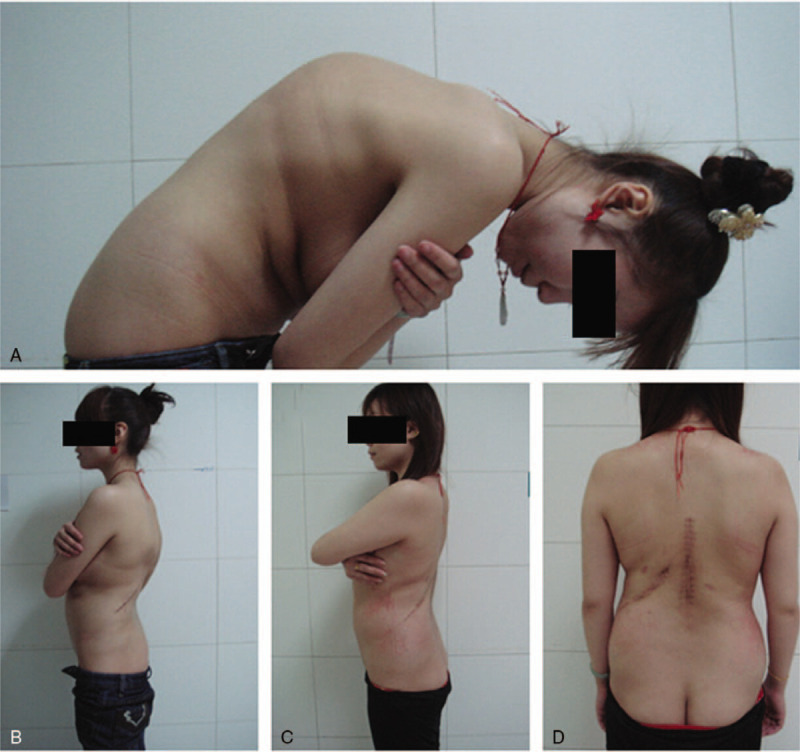
Photographs taken in clinic (A and B) preoperatively and (C and D) postoperatively to show the achieved cosmesis.

Every patient in the study was followed with radiological and physical evaluations to assess the correction and fusion for at least 18 months, with an average follow-up duration of 36.1 ± 13.2 months (range, 18–60 months). The follow-up radiographic and clinical data are provided in Table 2, and images for representative cases are presented in Figures 1–3. The average preoperative kyphosis angle of 54.6° ± 8.0° (range, 45°–68°) was corrected to 4.8° ± 1.5° (range, 3°–7°) immediately after the operation and to 6.8 ± 1.3° (range, 5°–9°) at the last follow-up. The difference was statistically significant (*t* = 19.49, *P* < .05). Radiographs confirmed that bony fusion was achieved in all patients at an average of 6.8 ± 1.2 months (range, 5–9 months) after revision surgery. All patients had satisfactory spinal sagittal and coronal balance at the last follow-up. None of our patients experienced implant failure. The average preoperative visual analog scale score of 6.2 ± 1.0 (range, 5–8) was reduced to 2.6 ± 0.5 (range, 2–3) at the last follow-up, showing marked improvement in patients’ pain. The difference was statistically significant (*t* = 11.78, *P* < .05). None of the patients suffered from any types of surgery-related neurological deterioration, and 1 patient whose neurological status was Frankel grade C preoperatively experienced an improvement to grade D. The mean preoperative Oswestry disability index of 39.8% ± 4.6% (range, 31%–48%) was significantly improved to 24.5% ± 4.7% (range, 15%–29%) at the final follow-up. The difference was statistically significant (*t* = 13.83, *P* < .05).

**Table 2:**
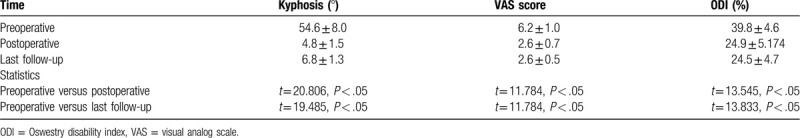
Radiographic and clinical data.

## 4 Discussion

### 4.1 Cause of failure

Inappropriate indications for an anterior approach may be the primary cause of failure of such treatment. In our series, patients with posttraumatic kyphosis exceeding 40° had been treated with an anterior approach alone in the initial surgery. Although some studies^[1,3,4]^ have reported successful treatment of posttraumatic kyphosis with anterior decompression and stabilization with anterior instrumentation, in their series, the deformities were of low grade, and the corrections were minor. For a long-established and rigid deformity, posteriorly contracted soft tissue and ankylosed facet joints hinder correction by means of anterior surgery alone. Without the release of posterior obstacles, anterior correction is unsatisfactory. High bending moments due to residual kyphosis are not eliminated, which increases stress on the interface between the strut and endplate, causing perforation of the endplate, subsidence, shift, and migration of the corporal screws and, ultimately, deformity progression. To prevent bone graft subsidence, adequate preparation of the endplate is also essential. In a biomechanical study, Oxland et al^[20]^ demonstrated that removal of the endplate significantly reduced local strength and stiffness, thus increasing the risk of implant subsidence. In some patients, such as those with spinal infection, more severe invasion will require more extensive debridement, which in turn can accelerate endplate destruction. Subsidence of the bone graft into the adjacent vertebral body through the endplate occurs when the endplate is destroyed by infection, and the tricortical iliac graft bone is stronger than the cancellous bone.^[21]^ With the occurrence of graft subsidence, the vertebral screws may toggle, shift, migrate, or even pull out. Two studies^[13,22]^ found that correction of the kyphosis angle and loss of correction were statistically significant in cases involving thoracic and thoracolumbar spinal tuberculosis, suggesting that anterior instrumentation is not efficacious for preventing correction loss in cases of spinal tuberculosis. Postsurgical deformities may also ensue from technical error. Malpositioning of the vertebral screws close to, or into, the intervertebral disc decreases the stiffness of the construct, thus increasing the likelihood of deformity progression. Other factors detrimental to bone healing and the ultimate fate of anterior constructs include advanced age, smoking, patient noncompliance, and reduced bone mineral density.

### 4.2 Treatment

Kyphosis can be corrected either by means of anterior instrumented fusion, posterior corrective osteotomy, or a combined anterior–posterior procedure. The use of anterior-only procedures for the correction of posttraumatic deformities and deformities in active spinal tuberculosis has been reported.^[1,3,4,23,24]^ The anterior approach in the case of revision is complicated by adhesions and fibrosis, both in the thoracic cavity and anterior to the posterior longitudinal ligament or dura, which necessitate complete clearance of the tissues before osteotomy. Thus, the procedure is difficult, is associated with considerable blood loss, and is time- and energy-consuming. Anterior osteotomy requires wider exposure with mobilization of the surrounding structures to provide good visualization and adequate working room for the surgeon, but altered local anatomy and widespread fibrosis increase the potential for inadvertent injury to the surrounding structures. Grossly distorted anatomy also makes localizing the spinal canal very difficult and thus increases the risk of neurological injury. Correction by means of purely opening up the anterior column can cause severe stretching of the spinal cord with corresponding risks of neurological deficits,^[25]^ and the anterior-only procedure cannot achieve satisfactory correction in such cases. In these revision cases, replacing the corporal screws is difficult because the previously instrumented vertebral bodies are often compromised and inadequate for repeated fixation. Additionally, it is not easy to extend anterior instrumentation with additional anchor points. The forceful distraction applied on the corporal screws may cause them to loosen, which ultimately results in correction loss. Due to the rigidity of this type of postsurgical deformity, circumferential osteotomy is required to increase the flexibility necessary for satisfactory correction. The affected segment should be completely removed, including the anterior apical fusion mass (including the collapsed or/and subsided bone graft, any residual vertebral body, and surrounding callus) and the corresponding posterior elements. This, in turn, necessitates more extensive osteotomy (vertebral column resection [VCR]). VCR shortens the posterior column and opens the collapsed anterior column adequately so that the deformity can be corrected without excessively shortening the spinal cord. This is facilitated by the insertion of an appropriately sized strut graft as anterior support. This procedure may be performed from an anterior–posterior or single posterior approach. Previous studies have reported the application of combined sequential anterior and posterior VCR for the treatment of severe rigid spinal deformities,^[26–29]^ but this combined approach is extremely taxing both for the patient and the surgeon, requiring a lengthy operation with a high risk of major complications. Furthermore, it is not possible to simultaneously control both the anterior and posterior columns.^[30–32]^ Great care must be taken to control the position of anterior reconstruction materials in anterior and posterior vertebral column resection because manipulation of the construct during posterior surgery may cause the anterior construct to move.^[33]^ To address these problems, we applied posterior vertebral column resection (PVCR) as revision surgery to manage rigid postsurgical deformities. In our series, the spine was approached both anteriorly and posteriorly, but the anterior approach was performed for the sole purpose of removing the internal implant. The osteotomy, correction of the deformity, and stabilization of the spine were performed using the posterior approach, which allowed for significant correction with minimal complications. PVCR can address deformities at the apex, simultaneously control both the anterior and posterior columns to prevent migration of the anterior graft, and facilitate controlled shortening across the resection gap.^[30–32]^ The advantages of this method include the direct and easy access to the apex of the deformity; the ability to release fibrosis and adhesions from the dural sac, which is imperative to ensure safe correction of the deformity; the ability to achieve circumferential decompression of the cord; and deformity correction under direct vision. Fusion can also be easily extended by adding more pedicle screws over more levels to achieve adequate fixation and evenly distribute axial loading, thus preventing implant loosening or correction loss and creating compressive force at the anterior strut graft inserted into the osteotomy site to maximize the healing potential. In the thoracolumbar spine, the anterior column is subject to great axial loads due to its straight shape at the transition zone. Restoration of normal alignment equalizes stress distribution and is beneficial for solid fusion and correction maintenance, and of course, the ultimate success of any construct or revision construct depends on successful bone fusion.^[34–36]^ Finally, use of the anterior approach solely for localized implant removal decreases the magnitude of the revision procedure and limits disturbances, risk of injury, and stretching of the anterior structures.

### 4.3 Pitfalls and complications

In revision surgery, the vascular and visceral structures are more prone to injury because they are entrapped in dense scar tissue and adhered to adjacent structures as well as the anterior surface of the spine. The complication rate following revision lumbar surgery is 3 to 5 times higher than that following a primary lumbar procedure.^[37]^ Left-sided implants are often in close proximity to the kidney, peritoneum, aorta, esophagus, and thoracic duct; thus, their position may be changed in the presence of significant postsurgical fibrosis and adhesions to adjacent structures. Before executing a repeated anterior approach, high-quality preoperative imaging should be obtained to appreciate the relationship of the anterior implants to the adjacent visceral and vessel structures. Once structures are identified intraoperatively, dissection should be performed into the scar tissue over and localized to the implant. Dissection should be kept to a minimum to reduce trauma and risk of injury to surrounding structures. Peritoneal and lung tears are most likely to occur during revision surgery. These should be repaired immediately to prevent enlargement. To minimize the risk of injury, careful handling of these structures, along with slow and meticulous dissection using blunt instruments, is required. During the osteotomy procedure, the surgeon must take care to always work within the bony confines and not stray into the soft tissue. As the final step of the osteotomy, osteoclasis of the lateral bony wall, especially in the previously instrumented side, decreases the risk of injury.

### 4.4 Limitations

Our study has several limitations. First, in this study, retrospective literature reviews were used to compare the efficacy of surgery, and no corresponding cases were established for comparison. The results showed that there was a certain subjective bias. Second, the small number of enrolled cases was another weakness. This is a study of revision surgery, and the indications for surgery need to be strictly grasped, so the number of registered cases was small and may have introduced selection bias. In addition, this study was a retrospective case study with a relatively short follow-up period and a lack of multicenter research results for further verification. In future work, we will increase the sample size and conduct longer-term follow-up to evaluate the clinical results through a prospective multicenter randomized controlled clinical trial.

In conclusion, for patients who have undergone prior anterior fusion with unacceptable deformity, salvaging rigid postsurgical kyphosis by means of anterior removal of the implant and PVCR is a safe but technically demanding procedure that should be performed by highly experienced surgical teams to achieve safe and satisfactory correction.

## Acknowledgment

The authors thank Dr Zheng Gong for English language editing of this paper.

## Author contributions

**Data curation:** Jian Lu, Zhe-Hao Dai.

**Resources:** Fei Chen, Yi-Jun Kang.

**Validation:** Hai-Sheng Li.

**Writing – original draft:** Jian Lu

**Writing – review & editing:** Fei Chen.
